# Acute and Subacute Toxicity and Cytotoxicity of *Opuntia Dillenii* (Ker-Gawl) Haw. Seed Oil and Its Impact on the Isolated Rat Diaphragm Glucose Absorption

**DOI:** 10.3390/molecules26082172

**Published:** 2021-04-09

**Authors:** Mohamed Bouhrim, Salima Boutahiri, Loubna Kharchoufa, Hamza Mechchate, Omkulthom Mohamed Al Kamaly, Ali Berraaouan, Bruno Eto, Abderrahim Ziyyat, Hassane Mekhfi, Abdelkhaleq Legssyer, Mohammed Aziz, Mohamed Bnouham

**Affiliations:** 1 Laboratory of Bioresources, Biotechnology, Ethnopharmacology and Health, Faculty of Sciences Mohammed First University, Oujda B.P. 717, Morocco; mohamed.bouhrim@gmail.com (M.B.); l.kharchoufa@ump.ac.ma (L.K.); a.berraaouan@gmail.com (A.B.); ziyyat@yahoo.fr (A.Z.); hmekhfi@yahoo.fr (H.M.); alegssyer@yahoo.fr (A.L.); azizmo5@yahoo.fr (M.A.); 2 Univ. Lille, University of Liège, University of Picardy Jules Verne, JUNIA, UMRT 1158 BioEcoAgro, Specialized Metabolites of Plant Origin, F-59000 Lille, France; boutahirisalima@gmail.com; 3 Research Team on the Chemistry of Bioactive Molecules and Environment, Laboratory of Innovative Materials and Biotechnology of Natural Resources, Faculty of Sciences, Moulay Ismaïl University, Meknes, Zitoune Meknes B.P. 11201, Morocco; 4 Laboratory of biotechnology, Environment, Agri-Food, and Health (LBEAS), Faculty of Sciences, Dhar El Mahraz, Sidi Mohamed Ben Abdellah University (USMBA), Fez B.P. 1796, Morocco; 5 Department of Pharmaceutical Sciences, College of Pharmacy, Princess Nourah Bint Abdulrahman University, Riyadh 11564, Saudi Arabia; 6 Laboratories—TBC, Faculty of Pharmaceutical and Biological Sciences, B.P. 83 Lille, France; titisfrance@gmail.com

**Keywords:** acute toxicity, subacute toxicity, cytotoxicity, glucose absorption, hemidiaphragm, *Opuntia dillenii*, medicinal plant, diabetes

## Abstract

This study aims to assess the safety of the *Opuntia dillenii* (Ker-Gawl) haw. seed oil (ODSO) and its effect on the glucose absorption activity of the isolated rat hemidiaphragm. This oil’s safety was studied by exploring its acute (doses 1, 3, 5, and 7 mL/kg) and subacute (doses 1 and 2 mL/kg) toxicities in albino mice and Wistar rats, respectively. The safety of the ODSO was also assessed by studying its effect on the HepG2 cell viability in vitro. The effect of ODSO, or combined with the insulin, on the glucose absorption activity of isolated rat hemidiaphragm was evaluated at the dose 1 g/L in vitro. The results demonstrated the safety of ODSO. Indeed, this study showed that this oil does not produce any mortality or signs of toxicity after the single-dose administration in mice. Additionally, the daily intake of the ODSO during four weeks does not induce a significant variation in the biochemical parameters and body weight of rats compared with the control group. Besides, the cell viability of HepG2 did not change in the presence of ODSO. On the other hand, the ODSO increased the glucose absorption activity of the isolated rat hemidiaphragm, and this activity was significantly enhanced when combined with insulin. This study confirms, on one side, the safety of this oil and its efficacy and, on the other side, encourages its potential use as a complement to treat diabetes.

## 1. Introduction

Prickly pear *Opuntia dillenii* (Ker-Gawl) Haw., located in the west and northeast Morocco, is a species from the Cactus family. Its fruit is eaten mostly in fresh fruit form, and it is used to control diabetes in folk medicine [1]. Many studies investigated the toxicological and biological effect of the pear, juice, and cladode of *O. dillenii*, but a few were interested in the seed oil’s nutritional benefit and biological activities. The analyzed seed oil’s components demonstrated that it contains considerable amounts of linoleic acid and sterols (unsaturated fatty acids), such as β-sitosterol and the presence of γ-tocopherol (vitamin E) [2]. Additionally, the oil is characterized by the presence of other substances such as phenols, well known for their health benefit [3,4]. Several kinds of research on this plant’s fruit seed oil have shown that it has numerous activities such as antioxidant [5], hepatoprotective [6], antilipidemic, antidiabetic, and antidiabetogenic [7,8].

Herbs have been commonly employed as the primary prevention method to treat diseases since ancient times [9,10]. Today, botanical medicine is becoming increasingly widespread worldwide, particularly in developing countries with a plethora of medicinal plants highly abundant, affordable, and accessible. Although the promising therapeutic potential of these plants’ use has been shown, there is still concern not only about their use but also about their safety [11]. In Moroccan culture, in particular, medicinal plants are commonly considered healthy or of low toxicity, because of their lengthy traditional usage [12,13,14].

Like medicines, extracts from medicinal plants have shown remarkable therapeutic potential, and they were proven not to be harmless products. The use of plants for therapeutic purposes must be made in a rational and controlled manner to avoid side effects that may even lead to the individual’s death. In this context, assessing the biological effects of herbal extracts must necessarily begin with the assessment of their general toxicity. Following the experimental process of preclinical studies, the evaluation of the general toxicity of a new substance (an extract in our case) is imperative in an acute manner, subacute and chronic, while varying the follow-up period and the frequency of administration of the doses to be tested [15].

Antidiabetic medicinal plants are not free from toxicity, as demonstrated in a study conducted by Bnouham et al. (2002), in which they have compiled a list of 94 species of antidiabetic plants, including 17 toxic species. Although the study indicates that toxicity is generally related to the administration route [16]. Diabetes mellitus is a chronic metabolic disturbance marked by a disorder in the metabolism of carbohydrates, proteins, and lipids, which leads to an increased blood sugar level. It is due to a failure in insulin secretion by the pancreas or peripheral cells’ response to the insulin effect. Insulin, which is a hypoglycemic hormone secreted by the β cells of Langerhans islets, is considered vital for controlling blood glucose concentration by facilitating glucose absorption and metabolism by peripheral tissues [17]. The long-term consequences of untreated diabetes mellitus may extend to microvascular and macrovascular complications (neuropathy, kidney disease, retinopathy, and vascular diseases). In the untreated state, people with diabetes are characterized by increased hepatic glucose production and decreased glucose absorption in adipose tissue and muscles, hence the need to treat and control this disease [17]. In a study by Bouhrim et al., the antidiabetic effect of *Opuntia dillenii* (Ker-Gawl) haw. seed oil (ODSO) is shown by its ability to regulate the disorder of biochemical parameters related to carbohydrate metabolism. ODSO showed a hypoglycemic effect during the four weeks of treatment in diabetic rats. In the same study, ODSO increased the level of hepatic glycogen and decreased glucosuria in this animal model of diabetes. Taking into account that the animal model of diabetes used in this study is characterized by partial destruction of pancreatic beta cells and not insulin resistance, it could be that ODSO stimulates peripheral tissues to store glucose or potentiates the effect of insulin [7]. Skeletal muscle is the central tissue to use blood glucose and the key focus tissue for insulin action. Insulin improves the skeletal muscle’s glucose uptake by raising the functional glucose transfer molecule (GLUT-4) in the plasma membrane [18]. For in vitro peripheral glucose uptake analysis, calculating glucose content in the rat hemidiaphragm is a commonly used and valuable technique [18]. This work aims to evaluate the safety of the ODSO and investigate its effect on the glucose absorption activity of the isolated rat hemidiaphragm to indicate a potential use in the treatment of diabetes.

## 2. Results

### 2.1. Acute Toxicity

The results of this acute toxicity test showed that ODSO is not toxic even at 7 mL/kg and also did not exhibit any signs of toxicity (diarrhea, vomiting, abnormal mobility, etc.) or mortality during the entire surveillance period.

### 2.2. Subacute Safety of Opuntia Dillenii Seed Oil

#### 2.2.1. Effect of ODSO on Hepatic Enzymatic Biomarkers (ALT, AST, and ALP)

**Figure 1:**
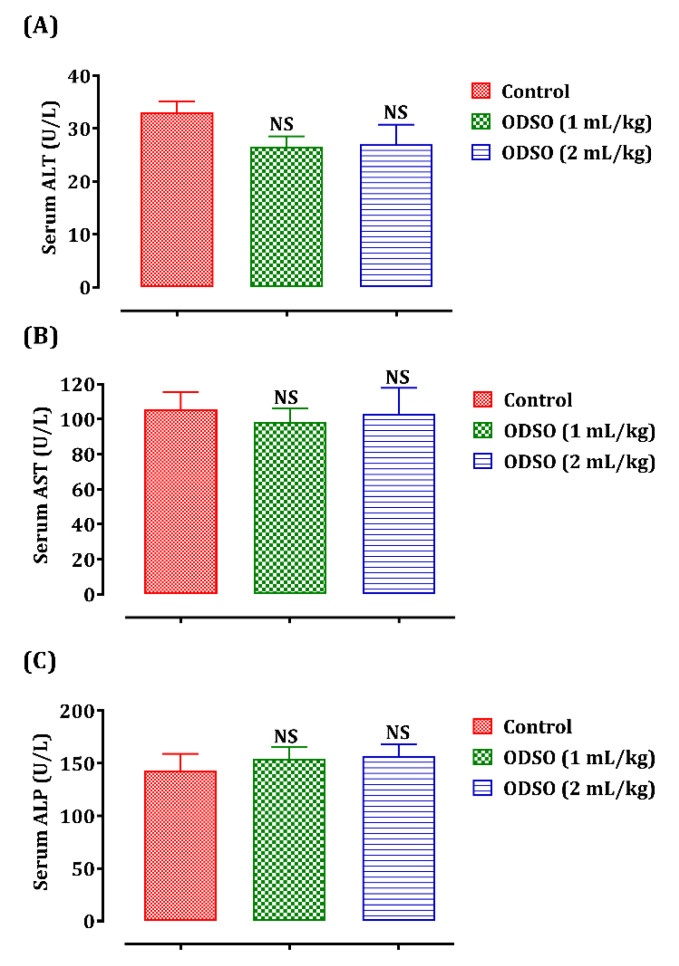
Oral administration effect of ODSO on ALT (**A**), AST (**B**), and ALP levels (**C**) in normal rats. Values are means (*n* = 6) ± SEM. NS: Not significant compared to the control, ODSO: *Opuntia dillenii* seeds oil; ALT: Alanine transaminase AST: aspartate aminotransferase ALP: alkaline phosphatase.

The daily ODSO administration effect during four weeks of treatment on liver function is shown in Figure 1. Daily administration of ODSO at a dose of 1 and 2 mL/kg during the 30 days did not cause any significant variation in the level of hepatic biomarkers, which are the hepatic transaminases alanine aminotransferase (ALT) (A), aspartate aminotransferase (AST) (B), and alkaline phosphatase (ALP) (C) in comparison with the control group.

#### 2.2.2. Effect of ODSO Administration on Plasma Bilirubin

**Figure 2:**
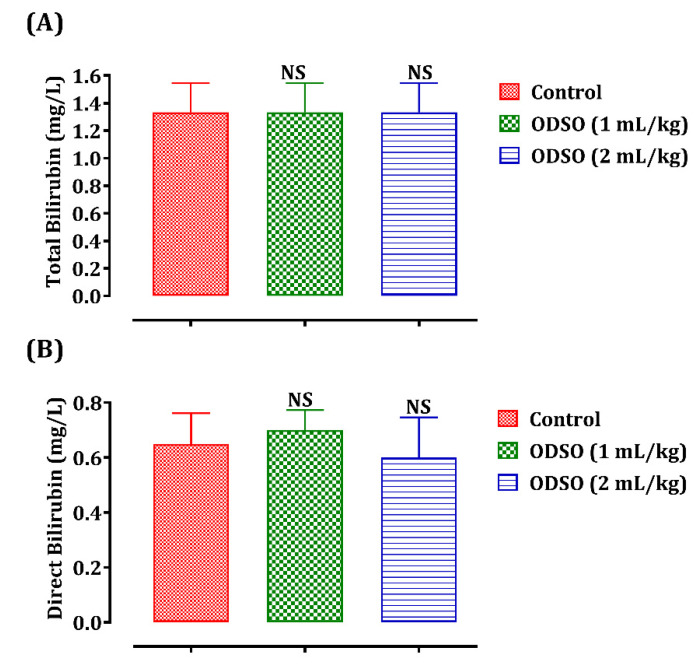
Oral administration effect of ODSO (1 and 2 mL) on total (**A**) and direct (**B**) bilirubin levels in normal rats. Values are means (*n* = 6) ± SEM. NS: Not significant compared to the control, ODSO: *Opuntia dillenii* seeds oil.

The variation in plasma bilirubin content depending on the administration of ODSO is shown in Figure 2. Indeed, the total (A) and direct (B) bilirubin levels in rats treated with ODSO (1 and 2 mL/kg) are similar to those in the control group.

#### 2.2.3. Effect of ODSO Administration on Lipid Profile

**Figure 3:**
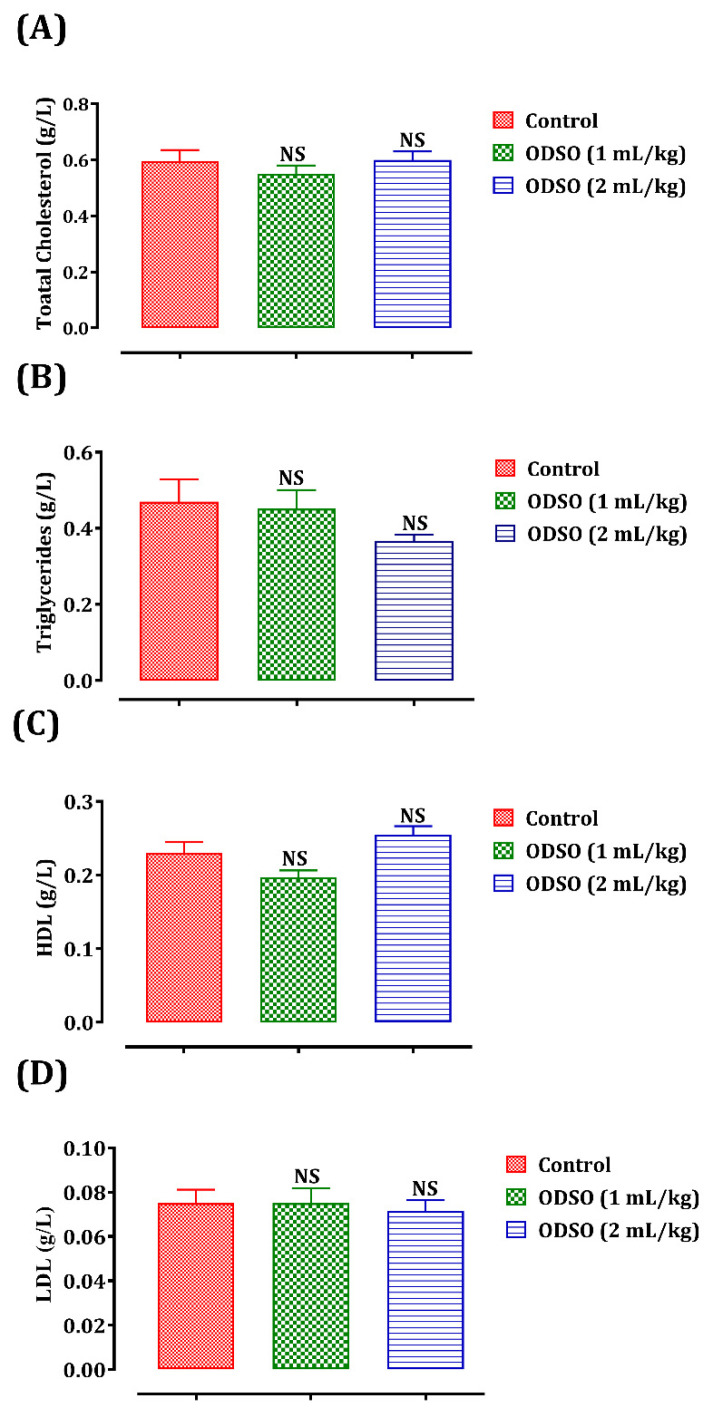
Oral administration effect of ODSO on plasma total cholesterol (**A**), triglycerides (**B**), HDL (**C**), and LDL (**D**) levels in normal rats. Values are means (*n* = 6) ± SEM. NS: Not significant in comparison with the control, ODSO: *Opuntia dillenii* seeds oil; HDL: High-density lipoprotein; LDL: Low-density lipoprotein.

The long-term administration of ODSO and its effect on rats’ lipid profile is shown in Figure 3. No significant changes were caused in plasma total cholesterol (A), triglyceride (B), HDL (High-density lipoprotein) (C), and LDL (Low-density lipoprotein) (D) levels in rats after daily administration of ODSO (1 and 2 mL/kg) compared to the untreated control group.

#### 2.2.4. Effect of ODSO on Total Protein and Blood Sugar

**Figure 4:**
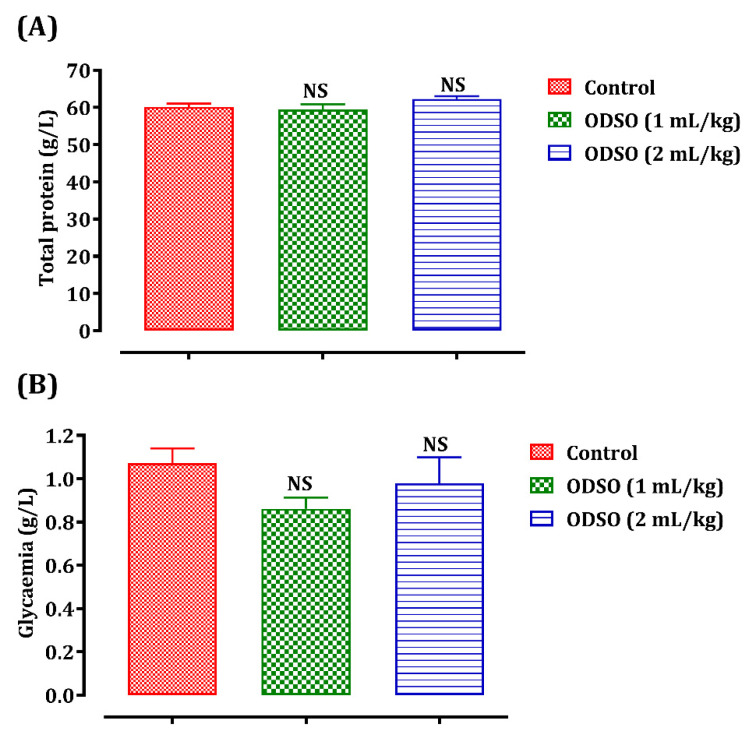
Oral administration effect of ODSO on the total protein (**A**) and blood sugar (**B**) levels in normal rats. Values are means (*n* = 6) ± SEM. NS: Not significant in comparison with the control, ODSO: *Opuntia dillenii* seeds oil.

Blood glucose and total plasma protein levels were also quantified to demonstrate the safety of ODSO in the long term (Figure 4). The daily gavage of rats with ODSO (1 and 2 mL/kg) for four weeks did not induce any significant variation in these two plasma parameters—total proteins (A) and blood sugar (B)—in comparison with the control group.

#### 2.2.5. Effect of ODSO on Renal Biomarkers (Uric Acid, Urea, and Creatinine)

**Figure 5:**
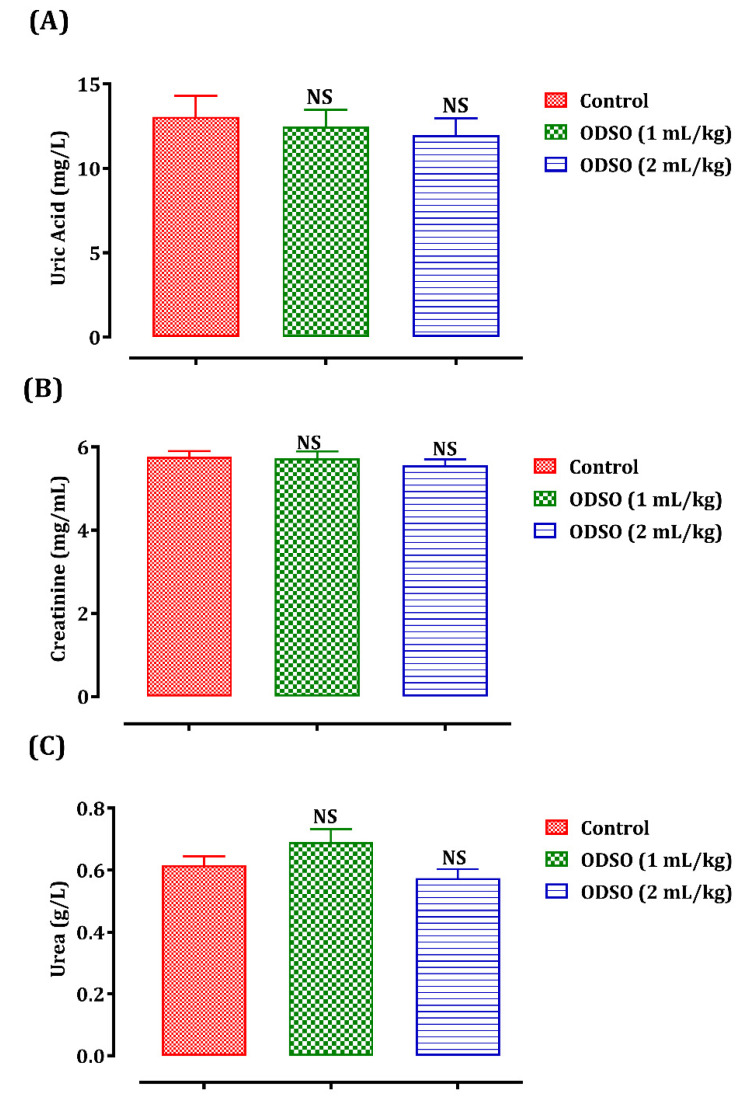
Oral administration effect of ODSO on the level of uric acid (**A**), creatinine (**B**), and uric acid (**C**) in normal rats. Values are means (*n* = 6) ± SEM. NS: Not significant in comparison with the control, ODSO: *Opuntia dillenii* seeds oil.

The biomarkers of renal function in the rats were explored after one month of ODSO administration (Figure 5). Daily administration of ODSO by normal rats and at doses of 1 and 2 mL/kg for 30 days did not influence the uric acid (A), creatinine (B), and uric acid levels (C) compared always to the control group.

#### 2.2.6. Effect of ODSO on Change in Body Weight

**Figure 6:**
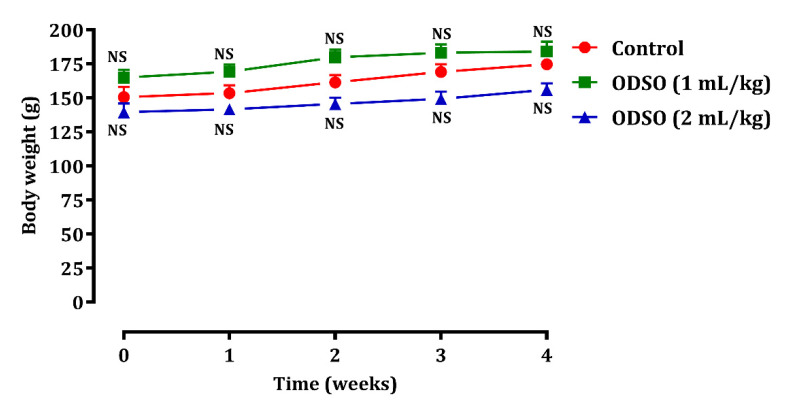
Oral administration effect of ODSO on change in body weight in normal rats. Values are means (*n* = 6) ± SEM. NS: Not significant in comparison with the control, ODSO: *Opuntia dillenii* seeds oil.

The safety of ODSO was also assessed by the change in body weight (Figure 6) during the four weeks of consumption of ODSO (1 and 2 mL/kg) in normal rats. The results showed that the change in body weight in the rats treated with ODSO was similar to that in the control group.

### 2.3. Effect of the Seed Oil of Opuntia Dillenii on HepG2 Cell Lines

**Figure 7:**
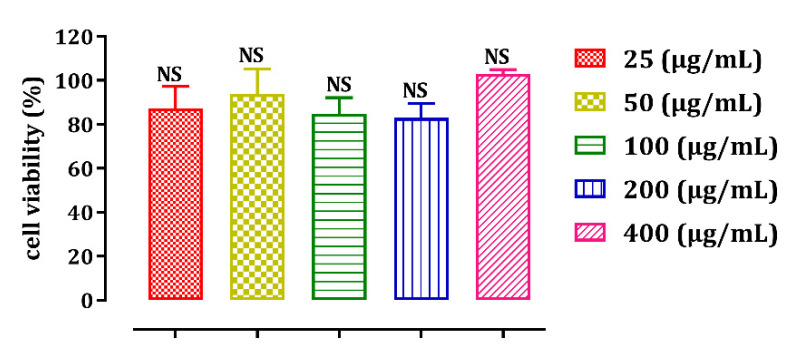
ODSO effect on the viability of HepG2 cells at different concentrations. Values are means ± SEM. NS: Not significant in comparison with other concentrations.

HepG2 cell lines were used in this study to predict the cytotoxicity of ODSO at various concentrations. (Figure 7). HepG2, the Human-derived liver cells has been widely used to predict mutagenicity, toxicity and carcinogenicity in humans [19]. After 48 h of incubation with various concentrations of the ODSO, the obtained results showed that cell viability stayed constant, in a dose-dependent way, with increasing concentrations of ODSO. The obtained IC_50_ was higher than the 400 µg/mL. Yosie et al. (2011) consider a substance safe when IC_50_ > 100 µg/mL, while Prayong et al. (2008) consider that a moderate cytotoxicity is when 100 µg/mL < IC_50_ < 1000 µg/mL, and a non-cytotoxicity is when IC_50_ > 1000 µg/mL [20,21]. Consequently, since no IC_50_ could be determined by the range of concentrations tested in this work, the ODSO is considered non-cytotoxic in HepG2 cell lines, according to Yosie and his collaborators. Moreover, since the highest concentration studied in this work was 400 µg/mL, no conclusion could be taken according to Prayong et al. model.

### 2.4. ODSO Stimulating Effect of Glucose Uptake by Isolated Rat Hemidiaphragm In Vitro

**Figure 8:**
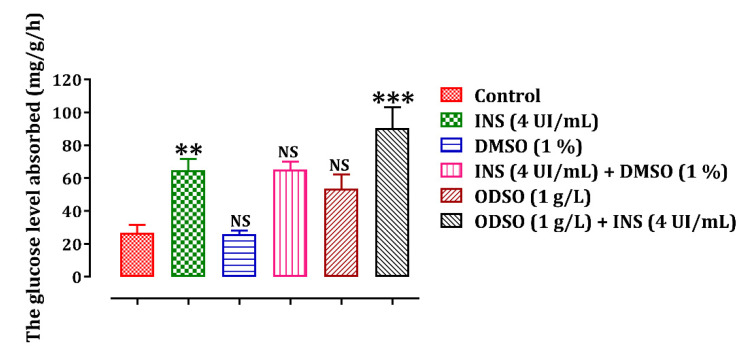
ODSO effect on glucose uptake by isolated rat hemidiaphragm. Values are means (*n* = 6) ± SEM. ** *p* < 0.01, *** *p* < 0.001: In comparison with the control group, NS: Not significant in comparison with the control, ODSO: *Opuntia dillenii* seeds oil; INS: insulin.

The incubation of the isolated rat hemidiaphragm in Tyrode’s solution and the presence of ODSO at a concentration of 1 g/L stimulated the uptake of glucose by the isolated rat hemidiaphragm (53.51 ± 8.84 mg/g/h) in comparison with the control group (26.64 ± 4.96 mg/g/h). Moreover, the incubation of the hemidiaphragm in the presence of ODSO (1 g/L) and insulin (4 IU/mL) significantly (*p* < 0.001) increased glucose absorption (90.50 ± 12.59 mg/g/h), compared to the incubated isolated rat hemidiaphragm, only in the presence of ODSO (Figure 8).

## 3. Discussion

In this study, we began with the acute toxicity test in Swiss albino mice using a dose range to determine essentially the lethal dose 50 (LD_50_), the maximum tolerable dose, and the observable-effect. After the test period, no mortality or apparent signs of toxicity were noted in treated animals. However, no histological assessments were done since we did not observe any toxicity signs. The absence of acute toxicity risk following the administration of a single dose of oil in mice does not indicate the absence of subacute toxicity. Therefore, the subacute toxicity of ODSO was evaluated in rats at doses of 1 and 2 mL/kg in the Wistar rat. This test showed that this antidiabetic plant is not toxic because it did not cause significant variations in biochemical parameters related to kidney and liver function and body weight in ODSO-treated rats. Besides, this oil’s safety was also evaluated by studying its effect on the HepG2 cell viability in vitro. The data from this test confirmed this oil’s safety, as cell viability was kept constant in the presence of this oil at increasing concentrations. To date, no studies have been conducted on the toxicity of ODSO. However, other studies have been conducted on other species of *Opuntia*. Indeed, in a study conducted by Berraaouan et al. (2014) to evaluate *Opuntia ficus-indica* seed oil’s toxicity in Albino Swiss mice, it was shown that it was not toxic. After the oral administration or intraperitoneal injection of the oil at various dose (1, 3, or 5 mL/kg), and during the 14-day treatment period, no deaths occurred and no abnormal behavioral or autonomous symptoms were found. [22]. In another study, low toxicity of the *Opuntia ficus-indica* seed oil was shown. The test showed that the lethal doses (LD_50_) are 43 and 2.72 mL/kg for the oral administration and the intraperitoneal injection, respectively [23]. From these studies, it can be concluded that the oil of *O. ficus-indica* seeds is not toxic even at high doses. Khémiri et al. (2019) evaluated the *O. ficus-indica* seeds oil’s acute toxicity in adult rats, revealing that the oil did not display any signs of toxicity or death in orally administered animals with either a 3.5 mL dose or a 7 mL oil/kg dose over the five days of the study period [24]. In this study, we also intended to examine the possible mechanism of action for ODSO’s antihyperglycemic effect [7], by evaluating this oil’s effect on the glucose absorption ability of the isolated rat hemidiaphragm. The measurement of glucose content in the rat hemidiaphragm is a standard and reliable procedure for studying peripheral glucose absorption in vitro [18]. This effect shows that the ODSO statistically augmented the glucose consumption of the isolated rat hemidiaphragm. In a study conducted by Haidara et al., the administration of γ-tocopherol (a compound present in the ODSO) increased the absorption of glucose into diaphragm muscles [25]. Another study showed that fish oil supplementation in the diet is well associated with a decrease in blood sugar via adjustments in the fatty acid composition of muscle membranes, which can directly or indirectly affect GLUT4 protein levels via regulation of the transcription or translation mechanism [26]. The content of unsaturated fatty acids was high in the ODSO. The sterol fraction was composed of β-sitosterol, campesterol, and fucosterol. For vitamin E, γ-tocopherol was present with a low amount [2]. Via a number of pathways, the fatty acid content of membranes may affect the behavior of membrane proteins involved in glucose transport. Borkman et al. demonstrated that the poly-fatty acid long-chain unsaturated phospholipid content of muscle membranes may affect insulin action through the effects of their physical properties on proteins such as insulin receptors and glucose transporters [27]. It has previously been demonstrated that diets high in polyunsaturated fatty acids promote glucose transport in cells and that improvements in the physicochemical microenvironments of lipid domain membranes correlated with glucose transporters influence insulin action. This modification can involve an improvement in muscle tissue membrane fluidity, which enhances or promotes the penetration of glucose carriers through the membrane. Fatty acids have been shown to regulate the glucose transport pathway physiologically [28]. A study conducted by Pu et al. demonstrated that acute exposure of L6 muscle cells to palmitic acid significantly stimulates glucose uptake via two distinct signaling pathways (PI3K/AMPK/Akt and PI3K/ERK), inducing an increase in translocated GLUT4 at the membrane surface [29]. However, the mixture of ODSO and insulin significantly augmented the glucose consumption activity of the isolated rat hemidiaphragm. In addition, these results agree with those of Saleh et al. which showed that olive oil supplementation to ovariectomized rats resulted in an enhancement of insulin-stimulated glucose uptake by diaphragm [30]. It appears that synergy may have occurred between ODSO and insulin when tested together. It can be concluded that ODSO improves hyperglycemia by an extra-pancreatic mechanism, and improves the effect of insulin on glucose absorption in the isolated rat hemidiaphragm [31]. Insulin has been documented to promote glucose transfer in the isolated rat diaphragm, mainly by translocating active glucose transport units from the intracellular membrane area to the plasma membrane [32]. Polyunsaturated fatty acids improved glucose capture after palmitic acid-induced insulin resistance in L6 muscle cells. Linoleic and α-linolenic acids strengthen glucose capture by C2C12 muscle cells that have been insulin resistant due to palmitic acid [33]. These results imply that the ODSO contains active compounds that, when combined with insulin at some concentration, enhance its influence. As a result, an improvement in glucose peripheral intake may be a factor involved in the antihyperglycemic behavior shown in the current research [34].

## 4. Materials and Methods

### 4.1. Chemicals, Reagents, and Materials

The following drugs and solvents were used in this study: D (+)-glucose anhydrous, which was purchased from Sigma Aldrich (Riedel-de Haen, Germany). Dimethyl-sulfoxide, sodium pentobarbital was purchased from Ceva Santé Animale (Paris, France), and glucose GOD-POD kit was purchased from Biosystems (Barcelone, Spain). Alanine aminotransferase (ALT), total bilirubin, direct bilirubin, aspartate aminotransferase (AST), alkaline phosphatase (ALP), total cholesterol, triglycerides, HDL, LDL, total protein, urea, creatinine, and uric acid kits were purchased from Biosystems, Spain. Insulin was purchased from the pharmacy (Cooper Pharma Company, Morocco). All chemicals were of analytical grade.

### 4.2. Collecting and Identifying Plant Material

In February 2018, *Opuntia dillenii* (Ker-Gawl) haw. freshly collected fruits from the region of Essaouira (Latitude: 31°30′45.00″ N Longitude: −9°46′12.00″ W), Morocco, were used as plant material in this study. Their identification was made by Professor Mohammed Fennan, a botanical expert from Mohammed V University’s scientific institute. The specimen was stored under the number HUMPOM 351 at the herbarium of the University of Mohammed First, Oujda, Morocco.

### 4.3. Preparation of the Seeds

The seeds were being separated from *O. dillenii* fruits after being mashed. They were well cleaned with distilled water and, after being air-dried, they were ground using an electric grinder to get a fine and homogeneous powder. This final powder was stored (−20 °C) until further use.

### 4.4. Seeds Oil Extraction

A total of 500 mL of petroleum ether was mixed to 100 g of the previously prepared seed powder to separate the seeds’ oil (repeated 2 times). The mixture was stirred for 24 h at room temperature. A rotary evaporator was used after filtration to get rid of the solvent. The oil was collected and stored at 4 °C.

### 4.5. Animals

This study was conducted on Swiss albino mice and Wistar rats (male and female). The animals were reared at the Department of Biology of the Faculty of Science of Oujda (a photoperiod of 12 h of light/12 h of darkness and temperature of 22 + 2 °C). The animals were kept under favorable conditions of rearing with free access to water and food.

### 4.6. Acute Toxicity

Acute toxicity was evaluated intraperitoneally on albino mice, weighing 22–32 g. Thirty mice have been regrouped into five lots (*n* = 6; ♂/♀ = 1), then treated by raising doses of ODSO (1, 3, 5, and 7 mL/kg) or distilled water (10 mL/kg). The signs of toxic effects and/or mortality have been observed after 2 h and every 24 h during 14 days after administration.

### 4.7. Subacute Toxicity Study of Opuntia Dillenii Seed Oil in Rats

#### 4.7.1. Grouping Rats

The normal rats weighing between 200 and 250 g were grouped into 3 groups (*n* = 6; ♂/♀ = 1). The control group received only the distilled water at the dose of 10 mL/kg, and the treated groups received the ODSO at the dose of 1 or 2 mL/kg.

#### 4.7.2. Test Procedure

During this study, the oil was administered to rats with oral gavage once a day for four weeks. Moreover, the bodyweight of the rats was measured weekly. At the end of this treatment period, the rats have fasted for 14 h. Then they were subjected to anesthesia, and the blood was collected by cardiac puncture and immediately centrifuged at 3000 rpm for 10 min. The plasma was then stored at −20 °C for biochemical analyses.

#### 4.7.3. Assays of Biochemical Parameters

Several biochemical parameters have been measured in plasma. Aspartate aminotransferase, alanine aminotransferase, alkaline phosphatase, bilirubin (total and direct), plasma glucose, total protein, urea, creatinine, uric acid, total cholesterol, triglycerides (TG), high-density lipoproteins (HDL-c), and low-density lipoproteins (LDL-c). All tests were performed with the COBAS INTEGRA^®^ 400-Plus analyzer, using standard clinical diagnostic kits.

### 4.8. Study of the Cytotoxicity of the Seed Oil of Opuntia dillenii

#### 4.8.1. Culture of Cell Lines

The human hepatoma HepG2 cell lines were used to evaluate the cytotoxic activity of the extract. The cells were grown in 75 cm^3^ flasks, containing Dulbecco’s modified Eagle medium (DMEM) culture medium supplemented with 10% fetal calf plasma (FCS) and antibiotics (a mixture of penicillin (100 U/mL) and streptomycin (100 U/mL)), at 37 °C in a sterile oven and a humid atmosphere saturated with 5% CO_2_. After incubation, the cell layer was rinsed with 10 mL of phosphate-buffered saline (37 °C), then bathed in 2 mL of trypsin ((0.5 mg/mL) + EDTA (0.2 mg/mL)). After trypsinization, the flasks were incubated at 37 °C for 4 to 5 min. The action of the trypsin was then stopped by adding 10 mL of supplemented culture medium.

#### 4.8.2. Evaluation of Cell Viability by the MTT Test

The HepG2 cells were diluted in the medium to have a seeding concentration of 3500 cells per well, then they were introduced into 96-well microplates (100 μL/well). After 48 h of incubation (37 °C, 5% CO_2_), the cells can be treated if they are in the exponential growth phase (between 80% and 90% confluence). The ODSO was solubilized in DMSO then diluted in a hot medium to have a concentration of 400 µg/mL. A series of dilutions were then carried out in microplates to obtain the following concentrations—400, 200, 100, 50, and 25 µg/mL. The culture medium is removed from the cell culture microplates and replaced with 100 μL of each dilution. Four repetitions were performed on each concentration tested, and a solvent control was also tested in four replications. The microplates were incubated for 48 h at 37 °C, 5% CO_2_. The medium was then removed and replaced with a solution of MTT at 0.5 mg/mL in complete DMEM medium (100 μL/well) for at least 1 h and 30 min at 37 °C. The MTT solution was then removed, and the insoluble formazan crystals formed at the bottom of the wells were dissolved in pure DMSO (100 μL/well). After a gentle stirring, the absorbance was measured at 550 nm. Knowing that the solvent control corresponds to 100% cell viability, the following formula was used to calculate the percentage of cell viability:

% Cell viability = (OD_avg_ cells treated/OD_avg_ control cells) × 100 ± SD

OD_avg_ cells treated: Average optical density of the 4 repetitions of each concentration.

OD_avg_ control cells: Average optical density of the 4 repetitions of the blank (solvent).

SD: Standard deviation.

### 4.9. Study of the Stimulatory Effect of the Absorption of Glucose by the Isolated Rat Hemidiaphragm

#### 4.9.1. Grouping of Animals

Wistar rats weighing between 200 and 300 g were used in this experiment. These rats were left on an empty stomach for 36 h before handling to consume all the glucose stored in the hemidiaphragm and therefore to avoid the release of glucose from the hemidiaphragm into the incubation medium. The rats were separated into 6 groups (*n* = 6; ♂/♀ = 1).

Control: Incubation in Tyrode’s glucose solution (1 g/L).

INS (4 IU/mL): Incubation in Tyrode’s glucose solution (1 g/L) + insulin (4 IU/mL).

DMSO (1%): Incubation in Tyrode’s glucose solution (1 g/L) + DMSO (1%).

INS (4 IU/mL) + DMSO (1%): Incubation in Tyrode’s glucose solution (1 g/L) + insulin (4 IU/mL) + DMSO (1%).

ODSO (1 g/L): Incubation in Tyrode’s glucose solution (1 g/L) + ODSO (1 g/L).

ODSO (1 g/L) + INS (4 IU/mL): Incubation in Tyrode’s glucose solution (1 g/L) + ODSO (1 g/L) + insulin (4 IU/mL).

#### 4.9.2. Experimental Design

Peripheral glucose uptake from rat hemidiaphragm was estimated according to the method described by Bnouham et al. [34]. Initially, the rat fasted 36 h were anesthetized slightly, followed by cervical dislocation. Once in total anesthesia (7–10 min), an incision at the abdominal wall is performed. Afterward, the hemidiaphragm is dissected, avoiding any trauma or destruction of this muscle. Then, the diaphragm is placed in a petri dish filled with cold, glucose-free Tyrode solution (NaCl, 134 mM; KCl, 2.68 mM; CaCl_2_, 1.80 mM; MgCl_2_, 1.05 mM; NaH_2_PO_4_, 417 μM; NaHCO_3_, 11.9 mM; Glucose, 5.56 mM) to wash it off any debris and cut it into two equal pieces. Immediately after cutting, each hemidiaphragm is placed in a tube filled with 10 mL of the Tyrode solution in which the product to be tested is solubilized. The tube is incubated in a married bath for 1 h and at 37 °C, leaving the continuous oxygenation passage (95% O_2_, 5% CO_2_) and under 120 cycles/min agitation. At the end of incubation, the isolated rat hemidiaphragm was removed, dried in the oven for 8 h (37 °C), and weighed (dry weight). The glucose contained in solutions was dosed using a commercial enzyme dosing kit (GLUCOSE, Biosystems) based on the GOD-POD method (Glucose oxidase and peroxidase). Glucose absorbed by the isolated rat hemidiaphragm is expressed in milligrams of glucose absorbed per gram of dry hemidiaphragm for one hour (mg/g/h).

### 4.10. Statistical Analyses

The study results were expressed as mean ± SEM, and the difference between the groups was calculated by one-way analysis of variance (ANOVA) using GraphPad Prism 7 for windows. The difference was considered significant when *p* < 0.05.

## 5. Conclusions

In conclusion, the toxicity investigation results confirm the safety of the *Opuntia dillenii* seed oil when used to treat diabetes. Moreover, the antihyperglycemic property of this oil can be thought an increase in glucose consumption by the diaphragm. Therefore, this oil could be beneficial to diabetic patients thanks to its hypoglycemic effect, to prevent or treat chronic hyperglycemia that causes the onset of diabetic complications. Lastly, further experiments are warranted to identify the active compounds and the mechanism of activity of this effect.

## Data Availability

Data are available upon reasonable request.

## Sample Availability

Samples of the compounds are available from the authors.
